# FDA committee unanimously recommends approval of dabigatran etexilate for stroke prevention in atrial fibrillation

**Published:** 2010-12

**Authors:** Aalbers J, Bryer A, Klug E

The US Food and Drug Administration (FDA) Cardiovascular and Renal Drugs Advisory Committee recently voted 9 to 0 in favour of recommending dabigatran etexilate for stroke prevention in patients with atrial fibrillation (AF).

For decades, vitamin K antagonists such as warfarin have been the most efficacious therapeutic option for stroke prevention in AF. Current recommendations for patients with non–valvular atrial fibrillation treated with warfarin recommend maintaining an international normalised ratio (INR) in the range of 2.0–3.0 through frequent blood monitoring and dose adjustments, which can be challenging for physicians and patients.

In RE–LY®, dabigatran etexilate demonstrated efficacy without the need for ongoing INR monitoring or dose adjustments. Furthermore, there were no food restrictions on those taking dabigatran in RE–LY®. A total of 6.3 million people in the USA, Japan, Germany, Italy, France, UK and Spain were living with AF in 2007 and this is expected to increase to 7.5 million by 2017, primarily due to the ageing population.^1^

‘We are pleased with the committee’s recommendation, which marks an important step in advancing care for patients with atrial fibrillation’, said Prof Klaus Dugi, Corporate Senior Vice President Medicine, Boehringer Ingelheim. ‘We believe dabigatran etexilate will offer patients and doctors the first new treatment option for stroke prevention in atrial fibrillation in more than 50 years. We look forward to working with the FDA as it finalises its review of dabigatran.’

 Pradaxa (75 and 110 mg) is currently only registered in South Africa for the prevention of venous thromboembolic events in patients who have undergone hip– and knee–replacement surgery. For full prescribing information refer to the package insert approved by the Medicines Regulatory Authority.
